# Glucose Metabolism in T Cells and Monocytes: New Perspectives in HIV Pathogenesis

**DOI:** 10.1016/j.ebiom.2016.02.012

**Published:** 2016-02-06

**Authors:** Clovis S. Palmer, Catherine L. Cherry, Isabel Sada-Ovalle, Amit Singh, Suzanne M. Crowe

**Affiliations:** a Centre for Biomedical Research, Burnet Institute, Melbourne, Australia; b Department of Infectious Diseases, Monash University, Melbourne, Australia; c Infectious Diseases Department, The Alfred Hospital, Melbourne, Australia; d School of Physiology, University of the Witwatersrand, Johannesburg, South Africa; e Instituto Nacional de Enfermedades Respiratorias Ismael Cosío Villegas, Mexico; f Department of Microbiology and Cell Biology, Centre for Infectious Disease and Research (CIDR), Indian Institute of Science, India

**Keywords:** HIV, Glucose transporter 1 (Glut1), Metabolism, Immunometabolism, HIV cure, HIV reservoir, Inflammation, Immune activation, Oxidative stress, CD4 T cells, CD8 T cells, Monocytes, Macrophage, SNAEs, Cardiovascular disease

## Abstract

Activation of the immune system occurs in response to the recognition of foreign antigens and receipt of optimal stimulatory signals by immune cells, a process that requires energy. Energy is also needed to support cellular growth, differentiation, proliferation, and effector functions of immune cells. In HIV-infected individuals, persistent viral replication, together with inflammatory stimuli contributes to chronic immune activation and oxidative stress. These conditions remain even in subjects with sustained virologic suppression on antiretroviral therapy. Here we highlight recent studies demonstrating the importance of metabolic pathways, particularly those involving glucose metabolism, in differentiation and maintenance of the activation states of T cells and monocytes. We also discuss how changes in the metabolic status of these cells may contribute to ongoing immune activation and inflammation in HIV- infected persons and how this may contribute to disease progression, establishment and persistence of the HIV reservoir, and the development of co-morbidities. We provide evidence that other viruses such as Epstein–Barr and Flu virus also disrupt the metabolic machinery of their host cells. Finally, we discuss how redox signaling mediated by oxidative stress may regulate metabolic responses in T cells and monocytes during HIV infection.

## Highlights

• Dysfunctional glucose metabolism in CD4+ T cells and monocytes is a hallmark of HIV+ infection.
• Increased glycolysis in CD4+ T cells in HIV+ individuals is associated with immune activation and low CD4+ T cell counts.
• Increased glycolytic flux is required for virion production and increases CD4+ T cell propensity to apoptosis.
• Glycolytic metabolites can disengage metabolism to control immune cell functions and promote survival of HIV-infected cells.
• Immunometabolism offers novel opportunities for HIV cure and remission strategies.
• Elevated Glut1 on pro-inflammatory monocyte is a potential biomarker for HIV-associated co-morbidities.

## 1 Introduction

Cells comprising the immune system are conditioned to respond rapidly and vigorously to antigenic and inflammatory signals, a process that heightens cellular metabolism and requires energy. Experiments using cell culture systems show that the glucose transporter Glut1, an integral membrane protein that mediates glucose uptake into the cell, is required for efficient HIV infection of CD4 + T cells (Loisel-Meyer et al., 2012) and is a marker of CD4 + T cell metabolic activity (Palmer et al., 2014a). Moreover, the number of CD4 + T cells that express Glut1 is increased in HIV-infected, treatment-naïve persons. This was reduced during suppressive antiretroviral therapy (ART) but was not completely normalized when compared to uninfected individuals. High circulating levels of these cells is associated with immune activation and a low CD4 + T cell count in patients, irrespective of treatment status (Palmer et al., 2014a). Macintyre and co-workers have demonstrated that Glut1 is required for CD4 + T cell activation and effector functions, and that Glut1 is critical for the transitioning from oxidative phosphorylation to aerobic glycolysis (Macintyre et al., 2014). Additionally, Oestreich et al., have recently shown that glycolysis is tightly controlled by the transcription repressor Bcl-6, which suppresses genes encoding molecules important in the glycolytic pathway in T cells (Oestreich et al., 2014). This demonstrates additional levels of metabolic control other than the canonical PI3K–Akt–mTOR axis, an intracellular signaling pathway that regulates glucose metabolism post-transcriptionally (Powell et al., 2011, MacIver et al., 2013, Palmer et al., 2015a).

Despite increasing awareness regarding the significance of HIV and growth cytokines in regulating glucose metabolism (Loisel-Meyer et al., 2012, Palmer et al., 2015b, Locasale and Cantley, 2011, Dagenais-Lussier et al., 2015), the physiological control of metabolism in immune cells in HIV infected persons has not been thoroughly investigated. However, new evidence indicates that intracellular redox state may exert metabolic control in T cells and monocytes during HIV infection (discussed in detail below). Indeed, it has long been proposed that augmented oxidative stress in HIV-infected individuals leads to accelerated disease progression, leading to suggestions that antioxidants might have a role in improving patients' health (Kotler, 1998). This review will discuss the recent advances in immunometabolism in the context of HIV and discusses how redox signaling may regulate key metabolic checkpoints in T cells and monocytes during HIV infection.

## 2 The Role of Glucose Metabolism in HIV Pathogenesis

### 2.1 Glucose Metabolism in CD4 + T Cells During HIV Infection

Data showing that CD4 + T cell metabolic activity is critical for HIV infectivity and immune activation is now gaining greater impact with increasing evidence that T cell activation shares an intimate association with metabolism (Hegedus et al., 2014). The well-established dogma is that activated CD4 + T cells are preferential targets for HIV infection. Activation of CD4 + T cells is accompanied by metabolic reprogramming which involves a switch from oxidative metabolism in resting cells to intensified glucose metabolism via aerobic glycolysis (Palmer and Crowe, 2012). Indeed, Macintyre and colleagues have revealed that Glut1 is essential for metabolic programming of CD4 + T cell activation, expansion and survival (Macintyre et al., 2014). Glycolysis results in the production of pyruvate from glucose with only a net of two adenosine triphosphates (ATPs) per molecule of glucose. By contrast if pyruvate proceeds through the tricarboxylic acid cycle (TCA cycle) to oxidative phosphorylation, an additional 36 ATP molecules are produced. This suggests a rather inefficient utilization of glucose, energetically, by activated CD4 + T cells which have a markedly increased demand for energy. Glycolysis also diverts the use of glucose for macromolecular biosynthesis essential for cell growth, proliferation, differentiation and maintenance of an activated state (MacIver et al., 2013). Distinct metabolic programming will also affect the levels of metabolites that can directly regulate immune cell functions (Loftus and Finlay, 2016).

The significance of glucose metabolic pathways in regulating HIV infection in CD4 + T cells has long been recognized. Early work by Sorbara et al. hinted at the significance of glucose metabolism in HIV infection by demonstrating that HIV infection of the H9 human T cell line induced the expression of Glut1 and Glut3 and increased glucose uptake (Sorbara et al., 1996). Further support came from Hollenbaugh and colleagues who found that HIV infection of primary CD4 + T cells in culture resulted in increased glucose uptake and expanded levels of glycolytic intermediates (Hollenbaugh et al., 2011). More link between glucose metabolism and HIV infection was established by Loisel-Myer et al., who showed that increased Glut1 expression on CD4 + T cells in culture increased cellular permissivity to HIV-1 infection, and that suppression of glucose metabolism by PI3K inhibitors inhibited infection (Loisel-Meyer et al., 2012). Although Loisel-Myer's group utilized LY294002, the non-selective isoform and non-tissue specific PI3K inhibitor, their results demonstrated that the PI3K pathway at least in part regulates glucose metabolism in CD4 + T cells. The PI3Kγ isoform has been proposed as potentially involved in glucose metabolic regulation in immune cells, but it's involvement in HIV infectivity is unclear (Palmer et al., 2015b). The significant relationship between metabolism in immune cells and HIV pathogenesis in humans has been elegantly reviewed recently by Dagenais-Lussier and co-workers. They proposed that dysregulated metabolism including excessive uptake of glucose by immune cells could induce hyper-immune activation which may result in HIV-related complications (Dagenais-Lussier et al., 2015).

### 2.2 Link Between Glucose Metabolism in T Cells and HIV Disease Progression

Inflammatory activation of T cells is accompanied by increased energy requirements via glycolysis, needed for the surge in cytokine production and differentiation into Th1 and Th17 cells. Evidence reveal that the glycolytic intermediate can directly control immune functions. It has been shown that the glycolytic metabolite phosphoenolpyruvate (PEP) is important in sustaining T cell receptor-mediated Ca^2 +^-NFAT signaling and effector functions (Ho et al., 2015). Although studies suggest that Glut1 is required for metabolic reprogramming of CD4 + T cell activation, its significance in HIV infected individuals and the impact on disease progression has only recently been studied (Palmer et al., 2014a). The pathogenesis of HIV disease is characterized by chronic immune activation, inflammation and increased oxidative stress. Even in the presence of effective antiretroviral therapy (ART), chronic immune activation persists and is associated with, and predictive of, incomplete CD4 + T cell recovery, as well as increased morbidity and mortality (Hunt et al., 2011a, Hunt et al., 2011b, Deeks, 2009, Fernandez et al., 2011). This persistent chronic immune activation places a high metabolic demand on CD4 + T cells in HIV infected individuals and cellular metabolic activity is increased in response to this imposed challenge.

In HIV infected patients there is an intricate immune balance and function of cellular immune subsets, such as regulatory T cells (Tregs) and T helper 17 (Th17) cells (Arruvito et al., 2012). Treg cells, characterized by Forkhead Box Protein 3 (Foxp3 +) expression, are an important subset capable of suppressing chronic immune activation and expansion of immune cells. Conversely, Th17 cells are characterized by IL-17 production allowing these cells to exert proinflammatory function against extracellular pathogens (Valverde-Villegas et al., 2015). CD4(+)CD25(+)Foxp3(+) Tregs have reduced ability to activate the PI3K/Akt pathway, a signaling pathway required for Glut1 expression and glucose metabolism (Palmer et al., 2015a). Human Tregs regulated by Foxo3 are unable to up-regulate Glut1 in response to T cell receptor ligation. Notably, pharmacologic activation of Akt overcomes the effects of Foxp3 in Tregs and induces Glut1 expression in these cells (Basu et al., 2015). In contrast, the proinflammatory Th17 cells, together with Th1 and Th2 cells express high surface levels of Glut1 and are highly glycolytic (MacIver et al., 2013, Palmer et al., 2015b, Michalek et al., 2011).

A sub-population of CD4 + T cells in humans that expresses Glut1 (CD4 + Glut1 +) was recently identified by flow cytometry using Glut1 antibody MAB1418 clone from R&D Systems, Minneapolis, Minnesota, USA (Palmer et al., 2014a). Although the reactivity of the antibody have been somewhat controversial (Kinet et al., 2007), it's specificity has been confirmed by Western Blot and flow cytometry in lentiviral-infected HEK293T cells overexpressing Glut1. Furthermore, intracellular Glut1 (Glut1_c-term_) analysis, using anti-Glut1 Ab40084 (Abcam) confirmed that glucose metabolism is increased in CD4 + T cells in HIV-infected persons (Palmer et al., 2014a). Other groups have used Glut1-RBD-GFP from Metafora Biosystems to study Glut1 expression on HIV infected primary CD4 + T cells (Hegedus et al., 2014). The percentage of circulating CD4 + Glut1 + cells is significantly increased in HIV-infected individuals and is a measure of persistent immune activation as well as being associated with a low CD4 + T cell count, irrespective of treatment status. Of note, the frequency of circulating CD4 + Glut1 + cells in long-term non-progressors is similar to those of treated virologically-suppressed individuals (Palmer et al., 2014a). These CD4 + Glut1 + T cells were highly activated as measured by the co-expression of CD38 and HLA-DR on their cell surface. Although an association between increased glucose metabolism, CD4 + T cell activation and low CD4 + T cell count has been established in HIV infection, the mechanisms underpinning these associations are unclear. It is worthwhile to note that protease inhibitors such as nelfinavir impair Akt signaling and Glut4 expression on adipocytes, and thus have been linked to Type 2 diabetes and insulin resistance (Kachko et al., 2009). Their effects on immune cell metabolism are less clear but have been shown to reduce anti-inflammatory gene expression in peripheral blood mononuclear cells (Yilmaz et al., 2010).

### 2.3 Metabolic Exhaustion — A Model for CD4 + T Cell Death in HIV -Infected Individuals

CD4 + T cell depletion during HIV infection occurs via two major pathways and has been reviewed (Cox and Siliciano, 2014). Productive infection of activated CD4 + T cells induces apoptosis, mediated by activation of both a DNA-dependent protein kinase (DNA-PK) and caspase-3 (Cooper et al., 2013). However, death of non-infected bystander CD4 + T cells occurs by an abortive infection-induced-pyroptosis, an IL-1β driven inflammatory process, mediated by caspase-1 activation (Doitsh et al., 2014). HIV-infected persons whose activated CD4 + T cells exhibit high glucose metabolic activities display elevated levels of immune activation and is associated with poor CD4 T cell recovery. These processes may be linked with high glucose metabolic activity having been shown to activate caspase-3, and IL-1β mediated caspase-1 expression in several cell types to induce cell death (Ho et al., 2000, Koenen et al., 2011). In untreated individuals, one potential mechanism by which HIV may dysregulate metabolism is through intracellular expression of Tat101 protein which suppresses the transcription of mtDNA, resulting in low ATP synthesis (Rodriguez-Mora et al., 2015).

**Fig. 1:**
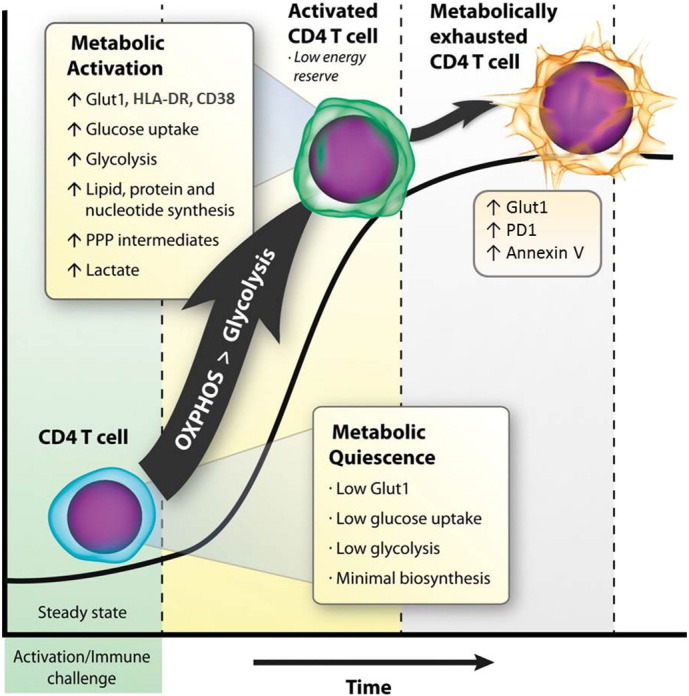
Model illustrating metabolic exhaustion of CD4 + T cells in HIV-infected individuals. Activation of CD4 + T cells is maintained by increased cell surface Glut1 expression and glycolysis. We propose a model whereby CD4 + T cells have a low energy reserve and therefore, prolonged activation results in metabolic exhaustion and cell death. PPP, pentose phosphate pathway.

In HIV + subjects effectively treated with ART, the percentage of circulating CD4 + Glut1 + T cells was more strongly associated with the absolute CD4 + T cell percentage than the classic markers of T cell activation, CD38 and HLA-DR (Palmer et al., 2014a). Furthermore CD4 + Glut1 + T cells expressed more PD1 and Annexin V, had higher levels of activation markers, and had increased glycolytic activity, compared with CD4 + Glut1 − cells (Palmer et al., 2014a). Consistent with a metabolically-exhausted phenotype, CD4 + Glut1 + T cells apoptose more rapidly in culture than CD4 + Glut1 − T cells (Palmer et al., 2013). Here we propose a model by which abnormally high glycolytic metabolism in CD4 + T cells during HIV infection fuels cell death. In this model we hypothesize that sustained activation of most CD4 effector T cells results in metabolic exhaustion and cell death for two main reasons, as follows. Firstly, activated CD4 + T cells have an inherently low energy reserve — they have a low spare respiratory capacity (SRC), which means that there is insufficient additional mitochondrial capacity available to maintain energy under increased stress or virus production. Thus compared to Tregs, Th1 effector cells have been shown to have lower SRC (Gerriets et al., 2015). Secondly, the high trafficking of ATP towards biosynthetic reactions (e.g. cell membrane and DNA synthesis) for CD4 + T cell growth and proliferation outpaces the amount of ATP generated by oxidative phosphorylation and glycolysis itself, thus resulting in metabolic exhaustion and cell death (Fig. 1). Supporting this model, Hegedus and colleague have demonstrated that glycolytic metabolism is essential for virion production and increases the sensitivity of infected primary CD4 + T cells to apoptosis. HIV-1 infected primary CD4 + T cells cultured in galactose have increased survival over those cultured in glucose and this was associated with reduced caspase 3 activation and apoptosis in cultures supplemented with galactose (Hegedus et al., 2014).

High-resolution CT and positron emission tomography (PET) scans have been used to evaluate thymic tissue in HIV aviraemic patients with poor recovery of CD4 + T cells. This technique involves injection of glucose tagged with a short-lived isotope (2-[18F]-fluoro-2-deoxy-d-glucose) ([18F]FDG), and so detect metabolically active tissue. Although FDG PET scans did not discover any metabolically active thymic tissue, there was detectable activity in the axillary, mediastinal, submandibular, upper cervical and/or hilar lymph nodes in some patients (Tanaskovic et al., 2011). More application of this technology may improve our understanding regarding how immune cell metabolism in tissues such as lymph nodes and arterial walls may contribute to HIV disease pathogenesis.

### 2.4 Role of T Cell Metabolism on HIV-associated Inflammation

The relationship between T cell metabolism and chronic inflammation during HIV infection is not well understood. An imbalance in the ratio of circulating levels between the pro-inflammatory Th1/Th17 and anti-inflammatory Th2 cells exists during HIV infection (Fanales-Belasio et al., 2009, Williams et al., 2013). Driven by the mTORC1 pathway and glycolysis, Th1 and Th17 cells maintain an activated state to secrete potent inflammatory cytokines including IFNγ, and TNF (Chang et al., 2013, Zheng et al., 2009, Delgoffe et al., 2011). These cytokines are well established mediators of chronic inflammation in HIV infected individuals.

Human Th17 cells represent a T cell subpopulation that expresses CCR6, IL-23R and is defined by production of IL-17, as well the lineage-defining transcription factor RORC2 in humans (Maggi et al., 2010). Th17 cells are enriched in the gut mucosa where they play a critical role in maintenance of the mucosal barrier and host defense against extracellular bacterial and fungal infections (Omenetti and Pizarro, 2015). Similarly to Th1 populations these cells are highly glycolytic. Th17 cell-inducing conditions induce glycolytic enzymes and increase glycolytic activity. Suppression of glycolysis inhibited Th17 development while promoting Treg cell differentiation. Furthermore, the transcription factor HIF-1α is selectively expressed in Th17 cells controlled by mTOR, a central regulator of glucose metabolism (Shi et al., 2011). Recent evidence has shown that uncommitted naive T cells have very high metabolic requirements for IL-17 inductions and is dependent on mTOR activity as the rate-limiting step (Kastirr et al., 2015).

Gerriets and colleagues conducted detailed in vivo and in vitro metabolic analysis of effector T cells (Teffs; Th1 and Th17) and Tregs (Gerriets et al., 2015). They demonstrated that inflammatory Teffs and CD4 + Tregs are characterized by distinct metabolic processes. In an in vivo model of active experimental autoimmune encephalomyelitis, Th17 cells had elevated levels of Glut1 in the spleen and inguinal lymph nodes, but not on FoxP3-expressing cells. Furthermore, both Glut1 and Glut3 were upregulated on Th17 but not Tregs in in vitro polarized CD4 + CD25 − T cells (Gerriets et al., 2015).

Although both Th1 and Th17 were highly glycolytic, Th17 cells had higher levels of glycolytic genes and intermediates, regulated by HIF-1α. On the other hand, glycolysis in Th1 cells appeared to be regulated posttranscriptionally without accumulation of glycolytic intermediates (Gerriets et al., 2015). Remarkably, suppression of glycolysis by 2DG prevented Teff proinflammatory cytokine production in vitro and inhibited the transcription factors T-bet and RORγT (Gerriets et al., 2015, Shi et al., 2011).

Unlike Th1 and Th17 cells, Tregs have greater respiratory capacity and metabolic plasticity and can metabolize lipids in addition to glucose. PDHK selectively regulates CD4 + T cell differentiation and inflammation and its inhibition specifically impairs Th17 while sparing Th1, and inducing Treg differentiation (Gerriets et al., 2015). Thus, inhibition of PDHK1 suppressed RORγT expression and IL-17 production in Th17 cells but had no effect on T-bet expression or IFN-γ production in Th1 cells (Gerriets et al., 2015).

Th17 cells have enhanced permissivity to HIV infection (Cecchinato et al., 2008, Alvarez et al., 2013), which raises the possibility that they could contribute to residual viral replication and inflammation in the gut of individuals on effective ART. IL-17 cytokines are induced by high glucose through involvement of key signaling pathways such as NF-κB, protein kinase-C, p38 mitogen activated protein kinase; leading to lymphocyte activation and adhesion to human umbilical vein endothelial cells (Kumar et al., 2014), relevant to the pathogenesis of HIV-associated complications like atherosclerosis (Maisa et al., 2015).

### 2.5 CD4 + T Cell Metabolism and Homeostatic Proliferation of HIV Reservoir

Glut1 expression on CD4 + T cells is essential for their proliferation and survival and is regulated by growth and inflammatory cytokines such as IL-2 and IL-7. A major HIV reservoir comprises memory CD4 + T cell subsets: central memory (CD45RA − CCR7 + CD27 +), effector memory (CD45RA − CCR7 − CD27 −) and transitional memory (CD45RA − CCR7 − CD27+ −) cells. These memory CD4 + T cells may harbor active reservoir (cells producing multiply spliced HIV RNA), latent reservoir (cells containing integrated HIV DNA), or defective provirus incapable of producing competent HIV virions. Homeostatic proliferation of these reservoirs creates a major barrier to HIV eradication. (Chomont et al., 2009).

Genetic characterization of HIV-1 DNA from memory T cells from peripheral blood and gut-associated lymphoid tissue (GALT) from eight HIV-infected patients after 4–12 years of suppressive cART suggests that continued low levels of virus replication is not the major cause of HIV persistence in these cell populations (Josefsson et al., 2013). The authors concluded that the presence of clonal intracellular HIV-1 sequences indicated that HIV-1-infected memory T cells homeostatically proliferate during suppressive therapy (Josefsson et al., 2013). Another study analyzing *env* and *pol* sequences generated following single-genome amplification of virus obtained from blood and sputum of six HIV-infected individuals during long-term suppressive cART reported that identical or monotypic HIV-1 DNA sequences increased over time during ART (Wagner et al., 2013), further suggesting that proliferation of cells harboring HIV provirus is a key mechanism in HIV-1 DNA persistence. Under physiological conditions, memory CD4 + T cells have low cell surface expression of Glut1 (Palmer et al., 2014a) and undergo slow turnover (basal homeostatic proliferation) (Purton et al., 2007), but can divide rapidly in the presence of inflammatory cytokines (acute homeostatic proliferation) (Frison et al., 2013). In HIV-infected persons, the percentage of circulating memory CD4 + T cells expressing Glut1 is elevated (Palmer et al., 2014a). It is plausible that high levels of cell survival cytokines such as IL-7, and persisting inflammation in HIV + ART-experienced patients keep memory CD4 + T cells in a metabolically primed glycolytic state, promoting additional rounds of proliferation and expanding the HIV reservoir. These discussions invite research design to establish whether suppression of homeostatic proliferation through targeting glucose metabolic pathways may be a feasible strategy to suppress or deplete the HIV reservoir (Palmer and Crowe, 2014a).

Although memory T cells have often been described as “resting”, a subset of these memory CD4 + T cells expresses intermediate levels of CD25, suggesting a basal level of cellular activation (Triplett et al., 2012). This is further supported by our observation that Glut1 level is significantly elevated on memory CD4 + T cell sub-populations in HIV-infected persons, irrespective of treatment status (Palmer et al., 2014a). No studies have directly investigated the impact of metabolic inhibitors on HIV reservoir size, but one investigation has provided proof-of-concept for potential roles of these drugs in HIV cure strategies. In an exploratory study evaluating the effect of the mTOR inhibitor sirolimus on HIV persistence in cART-treated HIV-infected kidney transplant recipients, Stock and colleagues showed that sirolimus was independently associated with lower levels of HIV DNA in CD4 + T cells (Stock et al., 2014) and suggested their data supported a controlled clinical trial to access the impact of this mTOR inhibitor on HIV persistence during effective ART (Stock et al., 2014).

### 2.6 Targeting CD4 + T Cell Metabolism in HIV Cure and Remission Strategies

The PI3K/Akt signaling pathway, a key regulator of glucose metabolism in immune cells has been shown to have a pivotal role in the maintenance of HIV-1 latency. A novel agonist of PI3K p110α, 1,2,9,10-tetramethoxy-7H-dibenzo[de,g]quinolin-7-one reactivated HIV in in vitro models of virus latency and increased HIV expression in CD8 +-depleted blood mononuclear cells from virally-suppressed HIV-infected persons on suppressive ART. Similarly, the histone deacetylase (HDAC) inhibitor vorinostat (SAHA) also reactivated HIV via activation of PI3K/Akt signaling pathway (Doyon et al., 2014).

In other work, Giacomet and colleagues showed an increased number of activated CD4 + and CD8 + T cells (CD25 +, HLA − DR +, CD69 +) in an infant with congenital HIV infection, who, after 3 years of age, despite repeatedly testing negative for HIV antibodies, HIV DNA, p24, and HIV RNA was not cured (Giacomet et al., 2014). CD4 + T cells enriched for Th1/17 polarized cells, which have been shown to be metabolically active under inflammatory conditions had elevated susceptibilities to HIV-1 (Gerriets et al., 2015, Sun et al., 2015). Furthermore it has been postulated that metabolically-active Glut1-expressing CD4 + T cells are potential targets for HIV (Loisel-Meyer et al., 2012). Macintyre and colleagues have shown that Glut1 cell surface expression and glycolytic metabolism is selectively essential for maintaining CD4 + T cells activation (Macintyre et al., 2014). Increased Glut1 expression and cellular metabolism may increase proliferation of HIV reservoir cells, and also enhance viral proliferation by providing ATP substrate for viral DNA replication, and metabolites for cellular survival and functions (Loftus and Finlay, 2016). Thus therapies to normalize metabolically active cells in scenarios where active HIV is limited but where activated CD4 + T cells still exist may provide opportunity for longer-term remission in virologically suppressed patients off ART.

**Fig. 2:**
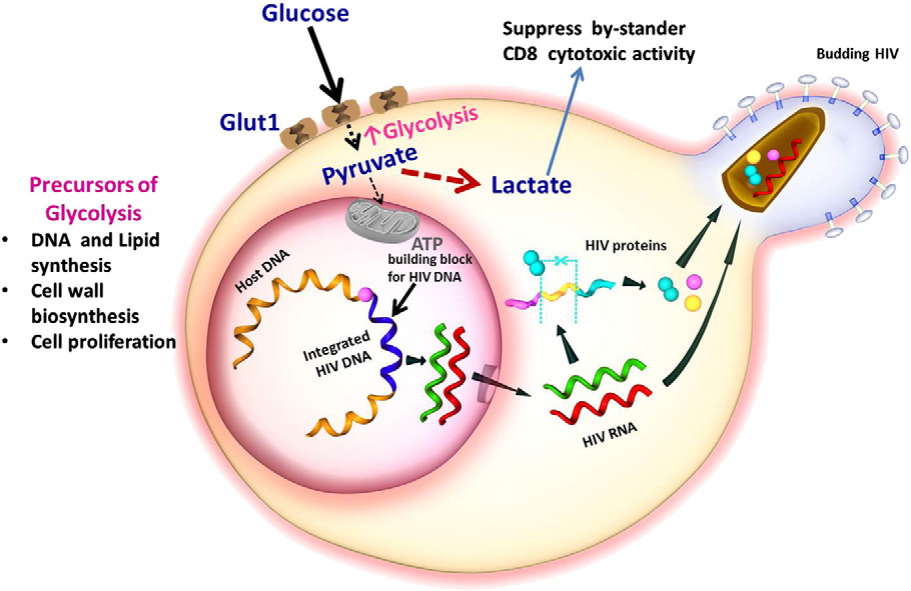
Increased Glut1 cell surface expression and glycolysis maintain CD4 + T cell activation. Suppression of the abnormally high levels of glycolysis in CD4 + T cells may be an approach to reduce activation and therefore limit the number of potential HIV targets.

In addition, lactate secreted as the end product of aerobic glycolysis by metabolically active CD4 + T cells, has been shown to suppress the proliferation and activity of cytotoxic T lymphocytes (Jones and Peterlin, 1994), which could subdue the ability of by-stander CD8 + T effector cells to counteract HIV in tissue compartments (Fig. 2) (Loisel-Meyer et al., 2012, Palmer and Crowe, 2012). Normalization of metabolic activity of CD4 + T cells may decrease the amount of HIV targets available in strategies utilizing the “shock and kill” procedure where CD4 + T cells may be inadvertently become activated (Rasmussen et al., 2014).

Indeed, it has been proposed that exploiting histone deacetylase inhibitors, such as vorinostat that disrupts HIV latency, with programmed interference in the metabolic stress of infected HIV cells is a potential strategy to purge the reservoir (Alonso-Villaverde et al., 2013). Metformin, a mitochondrial electron transport chain complex I inhibitor normalizes CD4 + T cell over-reactive metabolism (Yin et al., 2016, Yin et al., 2015). Metformin and chloroquine are potent modulators of pro-survival mechanisms to which HIV infected cells are challenged to generate energy to maintain virus production. While metformin may suppress cellular biosynthetic machinery by viral genes, chloroquine contributes to death of infected cells (Alonso-Villaverde et al., 2013). It raises the question whether combination of deacetylase inhibitors, metformin, and chloroquine during effective ART may be pursued to decay the HIV reservoir in patients (Alonso-Villaverde et al., 2013), however clinical evaluation of this strategy has not been conducted.

Metabolic-modifying drugs may also suppress HIV replication and spread independent of their immunomodulatory and metabolic properties. INK128 a dual ATP-competitive inhibitor of mTORC1 and mTORC2, suppress HIV-1 viremia in humanized mice by inhibiting the HIV lifecycle at the transcriptional level, while also suppressing CCR5 expression and R5 HIV entry into CD4 + T cells. Furthermore, INK128 boosted the antiviral potency of the CCR5 antagonist Maraviroc, and had favorable synergistic antiviral interactions with reverse transcriptase, integrase and protease inhibitors (Heredia et al., 2015). Adopting metabolic-modifying drugs that target cellular proteins necessary for successful completion of the HIV lifecycle have an advantage to slow emergence of resistance because cellular proteins have lower mutation rates than do viral proteins (Heredia et al., 2015).

### 2.7 Metabolic Targeting of the Macrophage HIV Reservoir

As demonstrated by GAPDH, which has posttranscriptional control over IFNγ expression in T cells (Chang et al., 2013), glycolytic enzymes do regulate immune cell functions and survival independent of canonical metabolic pathways. Hexokinase-1 (HK-1) a glycolytic enzyme that converts glucose to glucose-6-phosphate by aerobic glycolysis plays a ‘non-glycolytic’ role in the survival and maintenance of HIV-1- infected macrophages by binding to mitochondria and maintaining mitochondrial membrane potential. Disruption of the HK-1/mitochondrial interaction with a pharmacological agent, clotrimazole induced mitochondrial membrane depolarization, and caspase-3/7-mediated apoptosis (Sen et al., 2015). This suggests that manipulating this association in persistently infected macrophages may induce virus-associated cell death in HIV-infected macrophages (Sen et al., 2015).

It appears that HIV-1 hijacks the macrophage glucose metabolism machinery via Vpr-regulated HIF-1α to induce glycolytic genes such as HK-1, glucose-6-phosphate dehydrogenase, and pyruvate kinase muscle type 2 to promote viral replication and cellular biogenesis to prolong the survival of HIV-infected macrophages (Barrero et al., 2013). Thus strategies that disrupt transcriptional and posttranscriptional control of metabolic regulators may be exploited in combinatorial approaches for HIV eradication.

## 3 Metabolic Regulation of Monocytes

Monocytes dramatically change their metabolic phenotype from oxidative to glycolytic metabolism when activated in humans (Dietl et al., 2010, Cramer et al., 2003), similar to what is observed in T cells. In a study conducted by Vats and colleagues, classical activation of murine macrophages by IFNγ and lipopolysaccharide (LPS) strongly induced glucose uptake with a concomitant suppression of fatty acid uptake and oxidation (Vats et al., 2006). Evidence suggests that classically activated pro-inflammatory M1 macrophages demonstrate enhanced glycolytic metabolism and reduced mitochondrial activity. A similar metabolic profile represents the metabolic basis of “trained immunity” or “innate immune memory”, in which trained monocytes have increased aerobic glycolysis regulated through an Akt–mTOR–HIF-1α pathway (Cheng et al., 2014). In contrast, anti-inflammatory M2 macrophages are characterized by high mitochondrial oxidative phosphorylation, have enhanced spare respiratory capacity (Van den Bossche et al., 2015), and are reliant upon β-oxidation of fatty acids (Johnson et al., 2012, Mills and O'Neill, 2016). However, Hollenbaugh and colleagues more recently showed that while oxidative metabolism was increased, glucose uptake and glycolysis were reduced when the monocytic cell line U1 was infected with HIV (Hollenbaugh et al., 2011). This discrepancy is likely due to differences in activating stimuli and may relate to the use of cell lines as sources of monocytes, further complicated by the use of distinct monocytic cell lines U1 and U937 in their experiments. It is possible that, similar to CD4 + T cells, both metabolic programs are upregulated in monocytes and macrophages during activation, but glycolysis predominates. From a bioenergetics perspective, engaging oxidative phosphorylation maximizes ATP production so why would activated monocytes engage a less efficient energy generating pathway? It may be argued that engagement of glycolysis creates substrates for DNA and cell membrane synthesis to facilitate growth and differentiation of monocytes. Another possibility for the glycolytic shift by M1 macrophages is to facilitate optimal response suited for the rapid, short-term bursts of activation needed at sites of infection or inflammation as discrete metabolic patterns will result in varying levels of metabolites that can directly impact cellular function (O'Neill and Pearce, 2016). Citrate accumulation in the M1 macrophage is essential for the production of proinflammatory mediators such as NO, ROS, and prostaglandins (Infantino et al., 2011). In addition, metabolites and intermediates may serve as important signaling or regulatory molecules in immune cells. For example, the glycolytic enzyme GAPDH can bind to the AU-rich elements within the 3′ UTR of IFN-γ mRNA in T cells, and may disengage glycolysis and control effector cytokine production. Thus, aerobic glycolysis may also serve as a signaling mechanism to control inflammatory responses in immune cells (Chang et al., 2013).

### 3.1 Increased Glut1 Expression Supports Monocyte Differentiation and Macrophage Activation

Although differences in glucose metabolism are apparent between unactivated and activated monocytes (Palmer et al., 2014b), the metabolic signature of monocytes also varies depending on their differentiation state. The mechanisms governing the transition from activated monocytes to macrophage are poorly understood, but recent work by Malide et al., suggests that distinct temporal distribution and expression kinetics of glucose transporters characterize human monocyte differentiation to macrophages. Monocyte activation elicited by phorbol myristate acetate (PMA) stimulation resulted in Glut1 trafficking to the plasma membrane and Glut5 redistribution from the plasma membrane to intracellular compartments (Malide et al., 1998). In addition, LPS-stimulated murine macrophages showed enhanced glucose uptake which was attributed to expression of Glut1, the dominant glucose transporter in macrophages (Fukuzumi et al., 1996). In fact it is proposed that certain subpopulations of monocytes adopt a glycolytic profile allowing them to prolong their survival in low oxygen environments such as sites of chronic inflammation and tumors (Roiniotis et al., 2009).

### 3.2 Is There a Link Between Glucose Metabolism in Monocytes/Macrophages, Inflammation and HIV Infection?

**Fig. 3:**
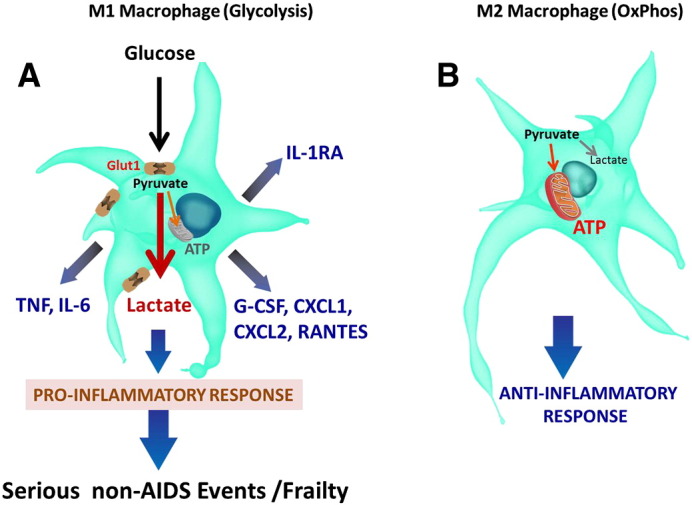
Glucose metabolic profiles of M1 and M2 macrophages. (A) Glucose metabolic profile of M1 macrophages. Classically activated macrophages induce aerobic glycolysis that results in lactate production and increased synthesis and secretion of inflammatory cytokines. (B) Glucose metabolic profile of M2 macrophages. Alternatively activated macrophages trigger a metabolic profile predominated by oxidative phosphorylation (OxPhos).

There are currently no established mechanistic links between increased glucose metabolism in monocytes/macrophages and inflammation in HIV-infected individuals. Freemerman and co-authors illustrated convincingly that Glut1 is the primary rate limiting glucose transporter on pro-inflammatory M1 macrophages (Freemerman et al., 2014). Macrophages that expressed Glut1 secreted high levels of pro-inflammatory mediators such as G-CSF, CXCL1, CXCL2, IL-6, RANTES, TNF and IL-1RA, and reactive oxygen species (Fig. 3) (Freemerman et al., 2014).

Independent work demonstrates that aerobic glycolysis positively regulates IL-6, IL1β and TNF mRNA expression in pro-inflammatory GM-CSF macrophages likely via HIF-1α/HIF-2α regulated genes (Izquierdo et al., 2015). Because the CCL2/CCR2 axis regulates monocyte recruitment to inflammatory sites (Sierra-Filardi et al., 2014) determining the metabolic factors that control their expression might identify novel targets for treating of HIV-associated co-morbidities such as atherosclerosis and cardiovascular disease, associated with inflammatory monocyte recruitment into inflamed blood vessels (Maisa et al., 2015). Since activated and pro-inflammatory monocytes play a critical role in HIV-associated co-morbidities, understanding the metabolic regulation of monocyte activation may provide novel therapeutic opportunities to improve the care of aging persons living with HIV.

**Fig. 4:**
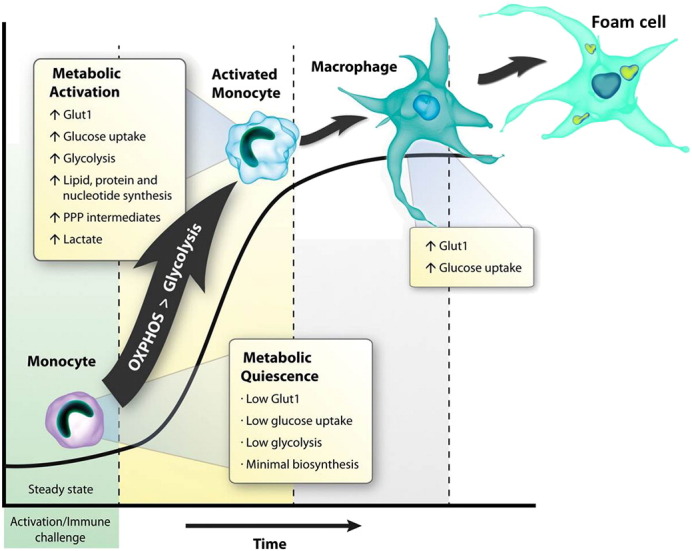
Monocyte metabolic profile changes during activation and differentiation. Monocytes display distinct metabolic profiles depending on their activation and differentiation states. Unactivated/naive monocytes are metabolically quiescent; their basal metabolic activity is low and ATP is derived primarily via oxidative phosphorylation (OxPhos). Upon activation/immune challenge monocytes shift to a state of metabolic activation characterized predominantly by Glucose transporter 1 expression (Glut1), increased glucose uptake, elevated glycolysis and biomass accumulation. Transition of monocytes to macrophage is characterized by further increases in Glut1 expression, glycolysis and biomass production. Activation of macrophages through ‘trained immunity’ may facilitate their transition to foam cells and instigate atherosclerosis.

We have shown that Glut1 is elevated on monocytes circulating in the blood of untreated and treated HIV-infected patients (Palmer et al., 2014b). Monocytes are currently classified into three major sub-populations based on the levels of CD14 and CD16 expression (classical: CD14 ++CD16 −, non-classical: CD14 + CD16 ++, intermediate: CD14 ++CD16 +). Compared to classical monocytes, non-classical and intermediate monocyte subsets have higher frequencies of Glut1-expressing cells. Higher Glut1 expression may be linked to a metabolic response to pro-inflammatory cytokine synthesis particularly within the intermediate monocyte population (Palmer et al., 2014b). This incites a scenario by which HIV infection (in treated and untreated individuals) in the setting of increased glucose metabolism and cellular activation provokes an inflammatory environment that increases the risk of age-associated co-morbidities. We have proposed a model in which monocyte activation during HIV infection facilitates a preferential shift in glucose metabolism from oxidative phosphorylation to aerobic glycolysis to sustain an inflammatory state with differentiation of macrophages into pro-atherogenic foam cells (Fig. 4) (Palmer and Crowe, 2014b). Although this explanation may have weight, experimental data will inform whether exploitation of drugs that selectively target glucose metabolic pathways in monocytes may be useful in the treatment of chronic inflammation in HIV-infected individuals.

The increased glycolysis observed during inflammatory activation of myeloid cells is accompanied by a surge in cytokine production and therefore pathways that regulate glucose metabolism are considered potential targets to treat inflammatory-mediated diseases. Several studies have highlighted the potential of metabolism-modifying drugs in treating inflammatory conditions. For example, activation of mTOR contributes to inflammatory-driven foam cell formation in patients with end-stage renal disease (Ma et al., 2014). Rapamycin has been suggested for the treatment of inflammatory-related diseases, including cardiovascular disease (Wullschleger et al., 2006), since it inhibits mTORC1 in macrophages (Ai et al., 2014). Using a cell-specific knockout approach Ai et al., established a regulatory role for mTORC1 in the suppression of inflammatory chemokines, CCl2 gene expression and atherogenesis (Ai et al., 2014). A large body of evidence indicate that other more specific mTORC1 inhibitors such as everolimus have pleiotropic anti-atherosclerotic effects making it a potential candidate as add-on therapy to prevent or delay the pathogenesis of atherosclerosis (Martinet et al., 2014).

## 4 Physiological Regulation of Glucose Metabolism: Oxidative Stress

### 4.1 Role of Redox Signaling in Modulating Inflammatory Responses During HIV Infection

Having described the importance of glucose metabolism in HIV-1 associated immune activation and inflammation, we would like to discuss a unique and underappreciated aspect of cellular physiology; that is, intracellular redox state in exerting genetic and metabolic control on HIV infection. Since energy metabolism is tightly coupled to changes in the intracellular redox state (Locasale and Cantley, 2011), we expect a functional linkage between immune activation, glucose metabolism and redox signaling in HIV-infected lymphocytes and macrophages. Earlier studies have shown an increase in reactive oxygen species (ROS) and a decrease in antioxidant levels (e.g., glutathione [GSH], thioredoxin [TRX]) in both HIV-infected cells and the plasma of asymptomatic patients during the early phase of the infection (Perl and Banki, 2000). Subsequently, it was found that redox signaling plays an important role in regulating gene transcription of HIV-1 through modulation of activity of redox-sensitive transcription factors such as NF-κB, AP-1, and p53 (Jones and Peterlin, 1994, Pereira et al., 2000), as well as regulation of cellular susceptibility of apoptosis (Banki et al., 1998). A continued increase in oxidative stress during the acute phase of HIV-1 infection ultimately results in the loss of mitochondrial membrane potential and induction of apoptosis (Banki et al., 1998). It appears that the low degree of oxidative stress is essential for initial replication of HIV-1, however, long-lasting oxidative stress at the later stages is required to infect bystander cells and promote apoptosis.

Studies have indicated that the perturbation in intracellular redox environment in HIV is linked to changes in proinflammatory cytokine and chemokine levels. For example, an increase in the levels of proinflammatory cytokines such as IL-1, TNF, and IL-17 in HIV-infected individuals correlated with an increased generation of free radicals (Morris et al., 2012). IL-1 in its receptor-bound form activates the expression of various other proinflammatory cytokines such as IL-6, IL-8 or TNF via the NF-κB system (Merrill et al., 1989). Further, both IL-1 and TNF production is mediated by either HIV infection or a physical association between gp120 and CD4 + molecules on mononuclear phagocytes (Merrill et al., 1989). Additionally, studies have shown a strong correlation between increased TGF-β and IL-1 production and a decrease in the expression of GSH biosynthetic genes and increased ROS production in macrophages of HIV-infected individuals (Morris et al., 2012, Franklin et al., 2003). Increased production of IL-17 in HIV-infected individuals may play a significant role in disease pathogenesis as it is known to induce the production of antimicrobial peptides and activate the secretion of IL-6, CCL-2, and TNF (Crome et al., 2010, Chang and Dong, 2007).

Abnormal levels of IL-17 may have both beneficial and adverse influence on HIV-infected patients. For example, high levels of IL-17 promote several inflammatory diseases such as asthma, multiple sclerosis, and arthritis, whereas low levels impair host defense against opportunistic pathogens such as *Mycobacterium tuberculosis* (*Mtb*) (Crome et al., 2010). TNF, another proinflammatory cytokine is increased in the serum of virally-suppressed HIV-infected persons and is associated with the proatherogenic phenotype of monocytes (Maisa et al., 2015). Increased generation of free radicals, oxidized lipids levels, and oxidized glutathione (GSSG) or decreased reduced glutathione (GSH) along with enhanced levels of proinflammatory cytokines, indicate that the breakdown in GSH homeostasis in HIV-infected individuals can be a result of chronic overproduction of inflammatory cytokines (Morris et al., 2012, Merrill et al., 1989). It is possible that chronic inflammation leads to an increased generation of free radicals, which leads to production of proinflammatory cytokines, which can further deplete GSH pool resulting in an overwhelming increase in ROS production. Until recently, the link between chronic inflammation and oxidative stress was not directly established. However, a recent study has shown that inflammatory macrophages release several GSH-coupled (glutathionylated) redox-active proteins. One of these glutathionylated proteins, peroxiredoxin-2 (PRDX-2), induces an oxidative cascade by triggering the production of TNF, thereby creating a self-promoting oxidative loop to induce further inflammation (Salzano et al., 2014). Interestingly, we have recently showed that HIV activation can modulate the expression of several GSH biosynthesis and peroxiredoxin genes (Bhaskar et al., 2015). These findings serve to indicate an important role of GSH homeostasis and inflammation in HIV pathogenesis.

### 4.2 Regulatory Dynamics Between Redox Homeostasis and Glucose Metabolism

To understand the importance of redox and inflammation, it is essential to understand how redox signaling is regulated during HIV infection. In this context, recent studies have indicated a direct role of central metabolism in controlling the redox state of HIV-infected cells. Studies have indicated an enhanced expression of glucose transporter Glut1 in HIV-1 infected H9 cells and in HIV-infected neuronal tissues (Sorbara et al., 1996, Kovitz and Morgello, 1997). Although no direct mechanistic link between oxidative stress and Glut1 expression exists in the context of HIV-infected cells, ROS production and evidence of increased oxidative stress have been demonstrated in macrophage-overexpressing Glut1 (Freemerman et al., 2014). Since HIV-infected cells display oxidative stress, it poses the question whether a direct link between glucose utilization and oxidative stress exists in HIV-infected cells. To protect against oxidative stress, the majority of cells exploit GSH as the major source of reducing equivalent under diverse patho-physiological conditions. However, it appears that de novo synthesis of GSH is compromised due to cysteine deficiency in HIV-infected patients (Nguyen et al., 2014). Consistent with this, intracellular levels of GSH were significantly diminished in both CD4 + and CD8 + T cells (Roederer et al., 1991). In addition, an increase in GSSG was also reported in HIV-1 infected CD4 + T-cells, indicating a general decrease in intracellular reductive capacity (Aukrust et al., 1995). Since regeneration of GSH will be dependent on the NADPH-mediated reduction of GSSG by GSH reductase (GR), the observed increase in glucose intake in HIV-infected donors could promote increase flux of glucose into the oxidative branch of the pentose phosphate pathway (PPP) to produce excess NADPH for GR-catalyzed GSSG reduction (discussed below).

At a regulatory level, glucose metabolism is controlled mainly by changes in Glut1 expression. Glut1 expression was found to be induced by oxidative stress (Andrisse et al., 2014), indicating that ROS can serve as a physiologically relevant signal to induce glucose metabolism of HIV-1 infected cells. Since high levels of glucose are known to induce oxidative stress in a variety of cells including lymphocytes, monocytes, and epithelial cells (Lan et al., 2013, Yao et al., 2006, Wu et al., 2009), it appears that regulatory mechanisms controlling the flux of glucose in PPP pathway to generate the necessary reductants (such as NADPH) for GSH-mediated detoxification of ROS might play a critical role in modulating the intracellular redox state to control HIV replication, persistence, and spread. The efficiency of NADPH production in the PPP cycle is dependent upon the expression of glucose-6-phosphate dehydrogenase (G6PD) and transaldolase (TAL). Over-expression of TAL reduces G6PD, NADPH, and GSH, rendering the cells susceptible to apoptosis induced by TNF, FAS signaling, and nitric oxide (NO), while repression of TAL activity inhibits apoptosis (Banki et al., 1996). Consistent with these findings, over-expression of TAL was shown to enhance HIV-induced oxidative stress and cell death in CD4^+^ T-cell lines, while repression had an opposing effect (Banki et al., 1996).

In addition to modulating intracellular redox environment, high glucose intake was found to enhance HIV entry into T cells by up-regulation of CXCR4 (Lan et al., 2013). Moreover, it has been shown that high glucose intake stimulated ROS generation in T cells, which induced the expression of CXCR4 by up-regulating HIF-1α, thereby resulted in enhanced HIV entry (Lan et al., 2013). Additionally, high glucose intake was shown to enhance the expression of proinflammatory cytokines such as TNF and IL-1β in monocytic cells. Both of these cytokines are known to increase HIV replication (Lan et al., 2013). Further, oxygen concentration and IL-7 levels appeared to modulate HIV infection by controlling Glut1 expression. Under physiologically relevant oxygen concentrations (5% oxygen tension), IL-7 stimulates HIV infection possibly through up-regulation of Glut1 (Loisel-Meyer et al., 2012). Since varying oxygen levels has a direct effect on GSH metabolism and intracellular ROS levels, it appears that IL-7 mediated regulation of Glut1 pathway and HIV permissiveness can also be under the control of the intracellular redox state of immune cells. However, the link between IL-7, _O_2 tension, Glut1 and redox needs further experimentation.

### 4.3 Dynamic Changes in Intracellular Glutathione Redox Potential (*E*_*GSH*_) Modulates HIV Persistence and Activation

The findings discussed above indicate that redox signaling is likely central to HIV infection and inflammation. However, no systematic, accurate and dynamic measurement of redox stress during HIV infection has yet been carried out. Working towards this goal, we have recently mapped the GSH redox potential (*E*_*GSH*_) of cytosol and mitochondria of HIV-infected cells during latency and replicative phases of infection (Bhaskar et al., 2014). Using a redox-sensitive GFP based non-invasive biosensor of *E*_*GSH*_, we demonstrated an increased capacity of latently infected monocytes and lymphocytes to tolerate oxidative insult and apoptosis (Bhaskar et al., 2014). Remarkably, we provided an accurate numerical indicator of *E*_*GSH*_ change required to reactivate virus from latency. We showed that a modest shift in *E*_*GSH*_ (25 mV) is sufficient to reactive HIV-1, suggesting the potential of purging HIV-1 reservoirs by redox modulators without adversely impacting cell survival (Bhaskar et al., 2014). Furthermore, we show that diverse agents of viral and bacterial origins such as HIV proteins Tat, and Nef, and lipids from *Mtb* activate HIV-1 and induce substantial oxidative stress in the cytosol and mitochondria of HIV-infected cells (Bhaskar et al., 2014). Our study suggests that the initial phase of viral replication resulted in oxidative stress from an increase in GSSG without significantly affecting the cellular ability to synthesize glutathione (GSH + GSSG). However, continuous viral replication results in an overwhelming oxidative stress, which is due to both decreased glutathione synthesis and compromised reduction of GSSG to GSH (Bhaskar et al., 2014). Lastly, our expression data demonstrated that cells latently infected with HIV-1 produce higher levels of antioxidants, whereas active viral replication is associated with oxidative stress (Bhaskar et al., 2014). Together, we provided the first quantitative assessment of the role of redox stress on HIV-1 persistence and replication.

According to our model, an increased expression of genes involved in glutathione biosynthesis and other cellular antioxidants endow cells with an enhanced capacity to resist oxidative stress and apoptosis during latency. However, a moderate oxidative shift in *E*_*GSH*_ can reactivate HIV-1 from persistence in latently infected cells. Active HIV-1 replication stimulated by viral proteins (Tat, Nef) or proinflammatory cytokines (TNF) can generate an excessive oxidative shift in *E*_*GSH*_, thereby promoting further increase in transcription from HIV-1 LTR through redox-dependent transcription factors such as NF-κB. These processes are likely central to the development of both AIDS and SNAEs in people with HIV infection. We envisage that our redox biosensor technology will be extremely useful in dissecting link between carbon metabolism, inflammation and redox signaling during HIV-1 infection. A more complete understanding between metabolic and immune processes will form the basis of future therapies to optimize the health of those living with HIV, and inform new strategies towards an HIV cure.

### 4.4 Immune Cell Glucose Metabolism in Other Disease Conditions

#### 4.1.1 Epstein–Barr Virus and Flu Virus

Epstein–Barr virus (EBV) is an oncogenic herpesvirus that exhibits a transient period of hyper-proliferation and a growth arrest phase that prevents outgrowth of infected cells. Recent work by McFadden and colleagues demonstrated that EBV-infected B-cells arrested after the early proliferation phase have reduced levels of mitochondrial respiration, decrease expression of genes TCA cycle genes, lower oxidative phosphorylation and reduced mTOR signaling. Notably, autophagy was also increased, a phenomenon important for cell survival and maintenance of early hyperproliferation during metabolic stress. On the contrary, long-term outgrowth was associated with increases glucose uptake and surface Glut1 levels, resulting in elevated glycolysis, oxidative phosphorylation, and suppressed basal autophagy (McFadden et al., 2016).

Although there is limited research evaluating the direct impact of viral infections on immune cell metabolism in humans, one recent study showed that intranasal vaccination of human volunteers with live influenza virus increased glycolysis in circulating plasmacytoid dendritic cells (pDCs). This metabolic pathway was shown to regulate pDC antiviral functions, including IFN-α production and phenotypic maturation, demonstrating for the first time the unrecognized role for metabolism in regulating pDC immune responses to viral infections in humans (Bajwa et al., 2016).

#### 4.1.2 Sepsis

As outlined above, efficient glucose influx is essential for optimal functions of monocytes and macrophages to support host responses to bacterial infection. Glucose transport via Glut1 by macrophages is required to promote ingestion of *Pseudomonas aeruginosa*, an important respiratory tract pathogen in individuals with cystic fibrosis (Barghouthi et al., 1995). In a mouse model of sepsis, increased glucose uptake and Glut1 expression occurred in macrophages following *P.* *aeruginosa* infection and LPS challenge (Gamelli et al., 1996).

## 5 Conclusion

Increased glucose metabolism in CD4 + T cells and monocytes is a hallmark of HIV infection and is driven by HIV proteins and inflammatory responses. This heightened metabolic state is regulated by Glut1 and required to maintain activation of CD4 + T cells, making them preferential targets for HIV infection and replication. Immune activation and reduced CD4 + T cell counts in HIV infected individuals are associated with increased expression of the glucose transporter Glut1 on CD4 + T cells. Increased glycolytic metabolism of monocytes is proposed as an underlying mechanism in the development of SNAEs such as frailty, cardiovascular complications and diabetes. We have highlighted the role of oxidative stress in HIV disease pathogenesis, noting that diminished levels of glutathione, a key mitochondrial antioxidant, is observed in HIV + individuals. Understanding the associations between metabolic dysfunction and oxidative stress in immune cells may provide an opportunity to involve antioxidant therapies in the care and management of HIV-associated co-morbidities (Nguyen et al., 2014).

There has recently also have been increased interest in the role of immunometabolism in the context of viral infections more generally. The concept of manipulating glycolysis and oxidative phosphorylation, a model already under investigation in cancer, to potentially impact immune cell survival and functions could be applied in the future development of novel potential forms of immunotherapy.

## 6 Outstanding Questions

Despite the evidence that increased glycolytic metabolism is an hallmark of pro-inflammatory monocytes and macrophages, a casual mechanistic link between metabolically active monocytes/macrophages and HIV-associated co-morbidities in HIV + individuals on suppressive ART has yet to be shown. Establishing such a link will provide novel opportunities to predict, diagnose, and treat a constellation of age-associated co-morbidities in HIV-infected individuals. Identifying the physiological inducers of dysfunctional immune cell metabolism, including understanding the relationship between oxidative stress and metabolism requires substantial work. The body of work reviewed here additionally raises questions of whether altering glucose metabolism in HIV reservoir cells may be an important facet of future HIV eradication strategies, and whether normalization of T cell metabolism can improve immune reconstitution and immunity in HIV-infected persons.

## 7 Search Strategy and Selection Criteria

We conducted a literature search of the PubMed electronic database for relevant articles; the key search terms used were “HIV metabolism”, “HIV Glut1”, “HIV T cell metabolism” “HIV monocyte metabolism” “HIV macrophage metabolism”, “Immunometabolism”, “HIV CD4 metabolism”, “HIV oxidative stress”, “Inflammation T cell metabolism”, and “Inflammation monocyte metabolism” We also conducted searches in different combinations without including HIV. Except for seminal articles, we included only those published between 2006 and February, 2016. Approximately 90% of referenced articles were published within this period, with 70% within the last 5 years.

## Author Contributions

C.S.P. conceptualized the review, organized, wrote the manuscripts, formulated the models, and designed the images. C.L.C. reviewed, edited and provided critical input. I.S. conducted literature search and edited the manuscript. A.S. wrote, reviewed, and edited the manuscript. S.M.C. reviewed, edited, and provided critical input.

## Disclosures

The authors have nothing to disclose.

## References

[bb0005] Ai D., Jiang H., Westerterp M., Murphy A.J., Wang M., Ganda A. (2014). Disruption of mammalian target of rapamycin complex 1 in macrophages decreases chemokine gene expression and atherosclerosis. Circ. Res..

[bb0010] Alonso-Villaverde C., Menendez J.A., Joven J. (2013). Metabolic stress in infected cells may represent a therapeutic target for human immunodeficiency virus infection. Med. Hypotheses.

[bb0015] Alvarez Y., Tuen M., Shen G., Nawaz F., Arthos J., Wolff M.J. (2013). Preferential HIV infection of CCR6 + Th17 cells is associated with higher levels of virus receptor expression and lack of CCR5 ligands. J. Virol..

[bb0020] Andrisse S., Koehler R.M., Chen J.E., Patel G.D., Vallurupalli V.R., Ratliff B.A. (2014). Role of GLUT1 in regulation of reactive oxygen species. Redox Biol..

[bb0025] Arruvito L., Sabatte J., Pandolfi J., Baz P., Billordo L.A., Lasala M.B. (2012). Analysis of suppressor and non-suppressor FOXP3 + T cells in HIV-1-infected patients. PLoS One.

[bb0030] Aukrust P., Svardal A.M., Muller F., Lunden B., Berge R.K., Ueland P.M. (1995). Increased levels of oxidized glutathione in CD4 + lymphocytes associated with disturbed intracellular redox balance in human immunodeficiency virus type 1 infection. Blood.

[bb0035] Bajwa G., DeBerardinis R.J., Shao B., Hall B., Farrar J.D., Gill M.A. (2016). Cutting edge: critical role of glycolysis in human plasmacytoid dendritic cell antiviral responses. J. Immunol..

[bb0040] Banki K., Hutter E., Colombo E., Gonchoroff N.J., Perl A. (1996). Glutathione levels and sensitivity to apoptosis are regulated by changes in transaldolase expression. J. Biol. Chem..

[bb0045] Banki K., Hutter E., Gonchoroff N.J., Perl A. (1998). Molecular ordering in HIV-induced apoptosis. Oxidative stress, activation of caspases, and cell survival are regulated by transaldolase. J. Biol. Chem..

[bb0050] Barghouthi S., Everett K.D., Speert D.P. (1995). Nonopsonic phagocytosis of *Pseudomonas aeruginosa* requires facilitated transport of d-glucose by macrophages. J. Immunol..

[bb0055] Barrero C.A., Datta P.K., Sen S., Deshmane S., Amini S., Khalili K. (2013). HIV-1 Vpr modulates macrophage metabolic pathways: a SILAC-based quantitative analysis. PLoS One.

[bb0060] Basu S., Hubbard B., Shevach E.M. (2015). Foxp3-mediated inhibition of Akt inhibits Glut1 (glucose transporter 1) expression in human T regulatory cells. J. Leukoc. Biol..

[bb0065] Bhaskar A., Munshi M., Khan S.Z., Fatima S., Arya R., Jameel S. (2014). Measuring glutathione redox potential of HIV-1 infected macrophages. J. Biol. Chem..

[bb0070] Bhaskar A., Munshi M., Khan S.Z., Fatima S., Arya R., Jameel S. (2015). Measuring glutathione redox potential of HIV-1-infected macrophages. J. Biol. Chem..

[bb0075] Cecchinato V., Trindade C.J., Laurence A., Heraud J.M., Brenchley J.M., Ferrari M.G. (2008). Altered balance between Th17 and Th1 cells at mucosal sites predicts AIDS progression in simian immunodeficiency virus-infected macaques. Mucosal Immunol..

[bb0080] Chang S.H., Dong C. (2007). A novel heterodimeric cytokine consisting of IL-17 and IL-17F regulates inflammatory responses. Cell Res..

[bb0085] Chang C.H., Curtis J.D., Maggi L.B., Faubert B., Villarino A.V., O'Sullivan D. (2013). Posttranscriptional control of T cell effector function by aerobic glycolysis. Cell.

[bb0090] Cheng S.C., Quintin J., Cramer R.A., Shepardson K.M., Saeed S., Kumar V. (2014). mTOR- and HIF-1alpha-mediated aerobic glycolysis as metabolic basis for trained immunity. Science.

[bb0095] Chomont N., El-Far M., Ancuta P., Trautmann L., Procopio F.A., Yassine-Diab B. (2009). HIV reservoir size and persistence are driven by T cell survival and homeostatic proliferation. Nat. Med..

[bb0100] Cooper A., Garcia M., Petrovas C., Yamamoto T., Koup R.A., Nabel G.J. (2013). HIV-1 causes CD4 cell death through DNA-dependent protein kinase during viral integration. Nature.

[bb0105] Cox A.L., Siliciano R.F. (2014). HIV: not-so-innocent bystanders. Nature.

[bb0110] Cramer T., Yamanishi Y., Clausen B.E., Forster I., Pawlinski R., Mackman N. (2003). HIF-1alpha is essential for myeloid cell-mediated inflammation. Cell.

[bb0115] Crome S.Q., Wang A.Y., Levings M.K. (2010). Translational mini-review series on Th17 cells: function and regulation of human T helper 17 cells in health and disease. Clin. Exp. Immunol..

[bb0120] Dagenais-Lussier X., Mouna A., Routy J.P., Tremblay C., Sekaly R.P., El-Far M. (2015). Current topics in HIV-1 pathogenesis: the emergence of deregulated immuno-metabolism in HIV-infected subjects. Cytokine Growth Factor Rev..

[bb0125] Deeks S.G. (2009). Immune dysfunction, inflammation, and accelerated aging in patients on antiretroviral therapy. Top. HIV Med..

[bb0130] Delgoffe G.M., Pollizzi K.N., Waickman A.T., Heikamp E., Meyers D.J., Horton M.R. (2011). The kinase mTOR regulates the differentiation of helper T cells through the selective activation of signaling by mTORC1 and mTORC2. Nat. Immunol..

[bb0135] Dietl K., Renner K., Dettmer K., Timischl B., Eberhart K., Dorn C. (2010). Lactic acid and acidification inhibit TNF secretion and glycolysis of human monocytes. J. Immunol..

[bb0140] Doitsh G., Galloway N.L., Geng X., Yang Z., Monroe K.M., Zepeda O. (2014). Cell death by pyroptosis drives CD4 T-cell depletion in HIV-1 infection. Nature.

[bb0145] Doyon G., Sobolewski M.D., Huber K., McMahon D., Mellors J.W., Sluis-Cremer N. (2014). Discovery of a small molecule agonist of phosphatidylinositol 3-kinase p110alpha that reactivates latent HIV-1. PLoS One.

[bb0150] Fanales-Belasio E., Moretti S., Fiorelli V., Tripiciano A., Pavone Cossut M.R., Scoglio A. (2009). HIV-1 Tat addresses dendritic cells to induce a predominant Th1-type adaptive immune response that appears prevalent in the asymptomatic stage of infection. J. Immunol..

[bb0155] Fernandez S., Tanaskovic S., Helbig K., Rajasuriar R., Kramski M., Murray J.M. (2011). CD4 + T-cell deficiency in HIV patients responding to antiretroviral therapy is associated with increased expression of interferon-stimulated genes in CD4 + T cells. J. Infect. Dis..

[bb0160] Franklin C.C., Rosenfeld-Franklin M.E., White C., Kavanagh T.J., Fausto N. (2003). TGFbeta1-induced suppression of glutathione antioxidant defenses in hepatocytes: caspase-dependent post-translational and caspase-independent transcriptional regulatory mechanisms. FASEB J..

[bb0165] Freemerman A.J., Johnson A.R., Sacks G.N., Milner J.J., Kirk E.L., Troester M.A. (2014). Metabolic reprogramming of macrophages: glucose transporter 1 (GLUT1)-mediated glucose metabolism drives a proinflammatory phenotype. J. Biol. Chem..

[bb0175] Frison H., Giono G., Thebault P., Fournier M., Labrecque N., Bijl J.J. (2013). Hoxb4 overexpression in CD4 memory phenotype T cells increases the central memory population upon homeostatic proliferation. PLoS One.

[bb0180] Fukuzumi M., Shinomiya H., Shimizu Y., Ohishi K., Utsumi S. (1996). Endotoxin-induced enhancement of glucose influx into murine peritoneal macrophages via GLUT1. Infect. Immun..

[bb0185] Gamelli R.L., Liu H., He L.K., Hofmann C.A. (1996). Augmentations of glucose uptake and glucose transporter-1 in macrophages following thermal injury and sepsis in mice. J. Leukoc. Biol..

[bb0190] Gerriets V.A., Kishton R.J., Nichols A.G., Macintyre A.N., Inoue M., Ilkayeva O. (2015). Metabolic programming and PDHK1 control CD4 + T cell subsets and inflammation. J. Clin. Invest..

[bb0195] Giacomet V., Trabattoni D., Zanchetta N., Biasin M., Gismondo M., Clerici M. (2014). No cure of HIV infection in a child despite early treatment and apparent viral clearance. Lancet.

[bb0200] Hegedus A., Kavanagh Williamson M., Huthoff H. (2014). HIV-1 pathogenicity and virion production are dependent on the metabolic phenotype of activated CD4 + T cells. Retrovirology.

[bb0210] Heredia A., Le N., Gartenhaus R.B., Sausville E., Medina-Moreno S., Zapata J.C. (2015). Targeting of mTOR catalytic site inhibits multiple steps of the HIV-1 lifecycle and suppresses HIV-1 viremia in humanized mice. Proc. Natl. Acad. Sci. U. S. A..

[bb0215] Ho F.M., Liu S.H., Liau C.S., Huang P.J., Lin-Shiau S.Y. (2000). High glucose-induced apoptosis in human endothelial cells is mediated by sequential activations of c-Jun NH(2)-terminal kinase and caspase-3. Circulation.

[bb0220] Ho P.C., Bihuniak J.D., Macintyre A.N., Staron M., Liu X., Amezquita R. (2015). Phosphoenolpyruvate is a metabolic checkpoint of anti-tumor T Cell responses. Cell.

[bb0225] Hollenbaugh J.A., Munger J., Kim B. (2011). Metabolite profiles of human immunodeficiency virus infected CD4 + T cells and macrophages using LC–MS/MS analysis. Virology.

[bb0230] Hunt P.W., Martin J.N., Sinclair E., Epling L., Teague J., Jacobson M.A. (2011). Valganciclovir reduces T cell activation in HIV-infected individuals with incomplete CD4 + T cell recovery on antiretroviral therapy. J. Infect. Dis..

[bb0235] Hunt P.W., Landay A.L., Sinclair E., Martinson J.A., Hatano H., Emu B. (2011). A low T regulatory cell response may contribute to both viral control and generalized immune activation in HIV controllers. PLoS One.

[bb0240] Infantino V., Convertini P., Cucci L., Panaro M.A., Di Noia M.A., Calvello R. (2011). The mitochondrial citrate carrier: a new player in inflammation. Biochem. J..

[bb0245] Izquierdo E., Cuevas V.D., Fernandez-Arroyo S., Riera-Borrull M., Orta-Zavalza E., Joven J. (2015). Reshaping of human macrophage polarization through modulation of glucose catabolic pathways. J. Immunol..

[bb0250] Johnson A.R., Milner J.J., Makowski L. (2012). The inflammation highway: metabolism accelerates inflammatory traffic in obesity. Immunol. Rev..

[bb0255] Jones K.A., Peterlin B.M. (1994). Control of RNA initiation and elongation at the HIV-1 promoter. Annu. Rev. Biochem..

[bb0260] Josefsson L., von Stockenstrom S., Faria N.R., Sinclair E., Bacchetti P., Killian M. (2013). The HIV-1 reservoir in eight patients on long-term suppressive antiretroviral therapy is stable with few genetic changes over time. Proc. Natl. Acad. Sci. U. S. A..

[bb0265] Kachko I., Maissel A., Mazor L., Ben-Romano R., Watson R.T., Hou J.C. (2009). Postreceptoral adipocyte insulin resistance induced by nelfinavir is caused by insensitivity of PKB/Akt to phosphatidylinositol-3,4,5-trisphosphate. Endocrinology.

[bb0270] Kastirr I., Crosti M., Maglie S., Paroni M., Steckel B., Moro M. (2015). Signal strength and metabolic requirements control cytokine-induced Th17 differentiation of uncommitted human T cells. J. Immunol..

[bb0275] Kinet S., Swainson L., Lavanya M., Mongellaz C., Montel-Hagen A., Craveiro M. (2007). Isolated receptor binding domains of HTLV-1 and HTLV-2 envelopes bind Glut-1 on activated CD4 + and CD8 + T cells. Retrovirology.

[bb0280] Koenen T.B., Stienstra R., van Tits L.J., de Graaf J., Stalenhoef A.F., Joosten L.A. (2011). Hyperglycemia activates caspase-1 and TXNIP-mediated IL-1beta transcription in human adipose tissue. Diabetes.

[bb0285] Kotler D.P. (1998). Antioxidant therapy and HIV infection: 1998. Am. J. Clin. Nutr..

[bb0290] Kovitz C.A., Morgello S. (1997). Cerebral glucose transporter expression in HIV infection. Acta Neuropathol..

[bb0295] Kumar P., Natarajan K., Shanmugam N. (2014). High glucose driven expression of pro-inflammatory cytokine and chemokine genes in lymphocytes: molecular mechanisms of IL-17 family gene expression. Cell. Signal..

[bb0300] Lan X., Cheng K., Chandel N., Lederman R., Jhaveri A., Husain M. (2013). High glucose enhances HIV entry into T cells through upregulation of CXCR4. J. Leukoc. Biol..

[bb0305] Locasale J.W., Cantley L.C. (2011). Metabolic flux and the regulation of mammalian cell growth. Cell Metab..

[bb0310] Loftus R.M., Finlay D.K. (2016). Immunometabolism: cellular metabolism turns immune regulator. J. Biol. Chem..

[bb0315] Loisel-Meyer S., Swainson L., Craveiro M., Oburoglu L., Mongellaz C., Costa C. (2012). Glut1-mediated glucose transport regulates HIV infection. Proc. Natl. Acad. Sci. U. S. A..

[bb0320] Ma K.L., Liu J., Gao M., Wang C.X., Ni J., Zhang Y. (2014). Activation of mTOR contributes to foam cell formation in the radial arteries of patients with end-stage renal disease. Clin. Nephrol..

[bb0325] Macintyre A.N., Gerriets V.A., Nichols A.G., Michalek R.D., Rudolph M.C., Deoliveira D. (2014). The glucose transporter Glut1 is selectively essential for CD4 T Cell activation and effector function. Cell Metab..

[bb0330] MacIver N.J., Michalek R.D., Rathmell J.C. (2013). Metabolic regulation of T lymphocytes. Annu. Rev. Immunol..

[bb0335] Maggi L., Santarlasci V., Capone M., Peired A., Frosali F., Crome S.Q. (2010). CD161 is a marker of all human IL-17-producing T-cell subsets and is induced by RORC. Eur. J. Immunol..

[bb0340] Maisa A., Hearps A.C., Angelovich T.A., Pereira C.F., Zhou J., Shi M.D. (2015). Monocytes from HIV-infected individuals show impaired cholesterol efflux and increased foam cell formation after transendothelial migration. AIDS.

[bb0345] Malide D., Davies-Hill T.M., Levine M., Simpson I.A. (1998). Distinct localization of GLUT-1, -3, and -5 in human monocyte-derived macrophages: effects of cell activation. Am. J. Physiol..

[bb0350] Martinet W., De Loof H., De Meyer G.R. (2014). mTOR inhibition: a promising strategy for stabilization of atherosclerotic plaques. Atherosclerosis.

[bb0355] McFadden K., Hafez A.Y., Kishton R., Messinger J.E., Nikitin P.A., Rathmell J.C. (2016). Metabolic stress is a barrier to Epstein–Barr virus-mediated B-cell immortalization. Proc. Natl. Acad. Sci. U. S. A..

[bb0360] Merrill J.E., Koyanagi Y., Chen I.S. (1989). Interleukin-1 and tumor necrosis factor alpha can be induced from mononuclear phagocytes by human immunodeficiency virus type 1 binding to the CD4 receptor. J. Virol..

[bb0365] Michalek R.D., Gerriets V.A., Jacobs S.R., Macintyre A.N., MacIver N.J., Mason E.F. (2011). Cutting edge: distinct glycolytic and lipid oxidative metabolic programs are essential for effector and regulatory CD4 + T cell subsets. J. Immunol..

[bb0370] Mills E.L., O'Neill L.A. (2016). Reprogramming mitochondrial metabolism in macrophages as an anti-inflammatory signal. Eur. J. Immunol..

[bb0375] Morris D., Guerra C., Donohue C., Oh H., Khurasany M., Venketaraman V. (2012). Unveiling the mechanisms for decreased glutathione in individuals with HIV infection. Clin. Dev. Immunol..

[bb0380] Nguyen D., Hsu J.W., Jahoor F., Sekhar R.V. (2014). Effect of increasing glutathione with cysteine and glycine supplementation on mitochondrial fuel oxidation, insulin sensitivity, and body composition in older HIV-infected patients. J. Clin. Endocrinol. Metab..

[bb0385] Oestreich K.J., Read K.A., Gilbertson S.E., Hough K.P., McDonald P.W., Krishnamoorthy V. (2014). Bcl-6 directly represses the gene program of the glycolysis pathway. Nat. Immunol..

[bb0390] Omenetti S., Pizarro T.T. (2015). The Treg/Th17 axis: a dynamic balance regulated by the gut microbiome. Front. Immunol..

[bb0395] O'Neill L.A., Pearce E.J. (2016). Immunometabolism governs dendritic cell and macrophage function. J. Exp. Med..

[bb0400] Palmer C.S., Crowe S.M. (2012). The role of glucose and lipid metabolism in the pathogenesis of HIV-1 infection. Curr. Trends Immunol..

[bb0405] Palmer C.S., Crowe S.M. (2014). Panobinostat clinical trial highlights the challenges towards an HIV cure. Lancet HIV.

[bb0410] Palmer C.S., Crowe S.M. (2014). How does monocyte metabolism impact inflammation and aging during chronic HIV infection?. AIDS Res. Hum. Retrovir..

[bb0415] Palmer C.S., Ostrowski M., Henstridge D.C., Lam L., Zhou J., Saleh S. (2013). Keystone Immune Activation in HIV Infection: Basic Mechanisms and Clinical Implications, Colorado, USA.

[bb0420] Palmer C.S., Ostrowski M., Gouillou M., Tsai L., Yu D., Zhou J. (2014). Increased glucose metabolic activity is associated with CD4 + T-cell activation and depletion during chronic HIV infection. AIDS.

[bb0425] Palmer C.S., Anzinger J.J., Zhou J., Gouillou M., Landay A., Jaworowski A. (2014). Glut1 expressing pro-inflammatory monocytes are elevated in cART-treated and untreated HIV-1 + subjects. J. Immunol..

[bb0430] Palmer C.S., Hussain T., Duette G., Weller T.J., Ostrowski M., Sada-Ovalle I. (2015). Regulators of glucose metabolism in CD4 and CD8 T cells. Int. Rev. Immunol..

[bb0435] Palmer C.S., Ostrowski M., Balderson B., Christian N., Crowe S.M. (2015). Glucose metabolism regulates T cell activation, differentiation, and functions. Front. Immunol..

[bb0440] Pereira L.A., Bentley K., Peeters A., Churchill M.J., Deacon N.J. (2000). A compilation of cellular transcription factor interactions with the HIV-1 LTR promoter. Nucleic Acids Res..

[bb0445] Perl A., Banki K. (2000). Genetic and metabolic control of the mitochondrial transmembrane potential and reactive oxygen intermediate production in HIV disease. Antioxid. Redox Signal..

[bb0450] Powell J.D., Pollizzi K.N., Heikamp E.B., Horton M.R. (2011). Regulation of immune responses by mTOR. Annu. Rev. Immunol..

[bb0455] Purton J.F., Tan J.T., Rubinstein M.P., Kim D.M., Sprent J., Surh C.D. (2007). Antiviral CD4 + memory T cells are IL-15 dependent. J. Exp. Med..

[bb0460] Rasmussen T.A., Tolstrup M., Brinkmann C.R., Olesen R., Erikstrup C., Solomon A. (2014). Panobinostat, a histone deacetylase inhibitor, for latent-virus reactivation in HIV-infected patients on suppressive antiretroviral therapy: a phase 1/2, single group, clinical trial. Lancet HIV.

[bb0465] Rodriguez-Mora S., Mateos E., Moran M., Martin M.A., Lopez J.A., Calvo E. (2015). Intracellular expression of Tat alters mitochondrial functions in T cells: a potential mechanism to understand mitochondrial damage during HIV-1 replication. Retrovirology.

[bb0470] Roederer M., Staal F.J., Osada H., Herzenberg L.A. (1991). CD4 and CD8 T cells with high intracellular glutathione levels are selectively lost as the HIV infection progresses. Int. Immunol..

[bb0475] Roiniotis J., Dinh H., Masendycz P., Turner A., Elsegood C.L., Scholz G.M. (2009). Hypoxia prolongs monocyte/macrophage survival and enhanced glycolysis is associated with their maturation under aerobic conditions. J. Immunol..

[bb0480] Salzano S., Checconi P., Hanschmann E.M., Lillig C.H., Bowler L.D., Chan P. (2014). Linkage of inflammation and oxidative stress via release of glutathionylated peroxiredoxin-2, which acts as a danger signal. Proc. Natl. Acad. Sci. U. S. A..

[bb0485] Sen S., Kaminiski R., Deshmane S., Langford D., Khalili K., Amini S. (2015). Role of hexokinase-1 in the survival of HIV-1-infected macrophages. Cell Cycle.

[bb0490] Shi L.Z., Wang R., Huang G., Vogel P., Neale G., Green D.R. (2011). HIF1alpha-dependent glycolytic pathway orchestrates a metabolic checkpoint for the differentiation of TH17 and Treg cells. J. Exp. Med..

[bb0495] Sierra-Filardi E., Nieto C., Dominguez-Soto A., Barroso R., Sanchez-Mateos P., Puig-Kroger A. (2014). CCL2 shapes macrophage polarization by GM-CSF and M-CSF: identification of CCL2/CCR2-dependent gene expression profile. J. Immunol..

[bb0500] Sorbara L.R., Maldarelli F., Chamoun G., Schilling B., Chokekijcahi S., Staudt L. (1996). Human immunodeficiency virus type 1 infection of H9 cells induces increased glucose transporter expression. J. Virol..

[bb0505] Stock P.G., Barin B., Hatano H., Rogers R.L., Roland M.E., Lee T.H. (2014). Reduction of HIV persistence following transplantation in HIV-infected kidney transplant recipients. Am. J. Transplant..

[bb0510] Sun H., Kim D., Li X., Kiselinova M., Ouyang Z., Vandekerckhove L. (2015). Th1/17 polarization of CD4 T Cells supports HIV-1 persistence during antiretroviral therapy. J. Virol..

[bb0515] Tanaskovic S., Fernandez S., French M.A., Price R.I., Song S., Robins P.D. (2011). Thymic tissue is not evident on high-resolution computed tomography and [(1)(8)F]fluoro-deoxy-glucose positron emission tomography scans of aviraemic HIV patients with poor recovery of CD4(+) T cells. AIDS.

[bb0520] Triplett T.A., Curti B.D., Bonafede P.R., Miller W.L., Walker E.B., Weinberg A.D. (2012). Defining a functionally distinct subset of human memory CD4 + T cells that are CD25POS and FOXP3NEG. Eur. J. Immunol..

[bb0525] Valverde-Villegas J.M., Matte M.C., de Medeiros R.M., Chies J.A. (2015). New insights about Treg and Th17 cells in HIV Infection and disease progression. J. Immunol. Res..

[bb0530] Van den Bossche J., Baardman J., de Winther M.P. (2015). Metabolic characterization of polarized M1 and M2 bone marrow-derived macrophages using real-time extracellular flux analysis. J. Vis. Exp..

[bb0535] Vats D., Mukundan L., Odegaard J.I., Zhang L., Smith K.L., Morel C.R. (2006). Oxidative metabolism and PGC-1beta attenuate macrophage-mediated inflammation. Cell Metab..

[bb0540] Wagner T.A., McKernan J.L., Tobin N.H., Tapia K.A., Mullins J.I., Frenkel L.M. (2013). An increasing proportion of monotypic HIV-1 DNA sequences during antiretroviral treatment suggests proliferation of HIV-infected cells. J. Virol..

[bb0545] Williams A., Steffens F., Reinecke C., Meyer D. (2013). The Th1/Th2/Th17 cytokine profile of HIV-infected individuals: a multivariate cytokinomics approach. Cytokine.

[bb0550] Wu C.H., Wu C.F., Huang H.W., Jao Y.C., Yen G.C. (2009). Naturally occurring flavonoids attenuate high glucose-induced expression of proinflammatory cytokines in human monocytic THP-1 cells. Mol. Nutr. Food Res..

[bb0555] Wullschleger S., Loewith R., Hall M.N. (2006). TOR signaling in growth and metabolism. Cell.

[bb0560] Yao K., Ge J.B., Sun A.J., Hong X.W., Shi H.Y., Huang R.C. (2006). Effects and mechanism of hyperglycemia on development and maturation and immune function of human monocyte derived dendritic cells. Zhonghua Xin Xue Guan Bing Za Zhi.

[bb0565] Yilmaz S., Boffito M., Collot-Teixeira S., De Lorenzo F., Waters L., Fletcher C. (2010). Investigation of low-dose ritonavir on human peripheral blood mononuclear cells using gene expression whole genome microarrays. Genomics.

[bb0570] Yin Y., Choi S.C., Xu Z., Perry D.J., Seay H., Croker B.P. (2015). Normalization of CD4 + T cell metabolism reverses lupus. Sci. Transl. Med..

[bb0575] Yin Y., Choi S.C., Xu Z., Zeumer L., Kanda N., Croker B.P. (2016). Glucose oxidation is critical for CD4 + T cell activation in a mouse model of systemic lupus erythematosus. J. Immunol..

[bb0580] Zheng Y., Delgoffe G.M., Meyer C.F., Chan W., Powell J.D., Anergic T. (2009). Cells are metabolically anergic. J. Immunol..

